# Model for End-Stage Liver Disease Score Predicts the Mortality of Patients with Coronary Heart Disease Who Underwent Percutaneous Coronary Intervention

**DOI:** 10.1155/2021/6401092

**Published:** 2021-04-17

**Authors:** You Chen, Min Han, Ying-Ying Zheng, Feng Zhu, Aikebai Aisan, Tunike Maheshati, Yi-Tong Ma, Xiang Xie

**Affiliations:** ^1^Department of Cardiology, First Affiliated Hospital of Xinjiang Medical University, Urumqi, China; ^2^Fuwai Central China Cardiovascular Hospital of Zhengzhou University, Department of Cardiology of Henan Provincial People's Hospital, Zhengzhou, China; ^3^Department of Cardiology, First Affiliated Hospital of Zhengzhou University, Zhengzhou, China

## Abstract

**Background:**

Coronary heart disease (CHD) is caused by the blockage or spasm of coronary arteries. Evidence shows that liver disease is related to CHD. However, the correlation between the Model for End-Stage Liver Disease (MELD) score and outcomes in patients after percutaneous coronary intervention (PCI) was unclear.

**Method:**

A retrospective cohort study involved 5373 patients with coronary heart disease after PCI was conducted from January 2008 to December 2016. Participants were classified to four groups according to the MELD score by quartiles. The primary endpoint was long-term mortality including all-case mortality (ACM) and cardiac mortality (CM). Secondary endpoints included bleeding events, readmission, major adverse cardiovascular events (MACE), major adverse cardiovascular, and cerebrovascular events (MACCE). The longest follow-up time was almost 10 years.

**Results:**

There were significant differences in the incidences of ACM (*p*=0.038) and CM (*p*=0.027) among the four MELD groups, but there was no significant difference in MACEs (*p*=0.496), MACCEs (*p*=0.234), readmission (*p*=0.684), and bleeding events (*p*=0.232). After adjusting the age, gender, smoking, drinking status, and diabetes by a multivariable Cox regression analysis, MELD remains independently associated with ACM (HR:1.57, 95%CI 1.052–2.354, *p*=0.027) and CM (HR:1.434, 95% CI 1.003–2.050, *p*=0.048).

**Conclusion:**

This study indicated that the MELD score had a strong prediction for long-term mortality in CHD patients who underwent PCI.

## 1. Introduction

Coronary heart disease (CHD) is caused by stenosis or obstruction of coronary atherosclerosis, which leads to high morbidity and mortality and seriously threatens the global health [1, 2]. Percutaneous coronary intervention (PCI) is the optimal strategy for the CHD, and thus, the proper evaluation of prognosis in patients after PCI is urgently needed. Recently, a large number of new models have been used to predict the clinical outcomes after PCI [3], but are still far from optimal.

The Model for End-stage Liver Disease (MELD) score including serum creatinine (sCr), total bilirubin (TB), and international normalized ratio (INR) is commonly used to estimate the prognosis in patients with chronic liver diseases [4]. Recently, it is reported that the MELD score can effectively predict the outcomes in patients with severe liver disease who undergone cardiac surgery or PCI [5, 6]. Additionally, studies show that sCr and INR have a good predictive effect on the long-term prognosis in patients after PCI [7, 8]. Kiris et al. [9] demonstrate that the MELD score combined with left ventricular ejection fraction could predict the mortality in patients with acute coronary syndrome (ACS) undergoing PCI, and the higher MELD score shows a higher rate of cardiac death.

However, there is no report on the correlation between the MELD score and prognosis in CHD patients after PCI. Therefore, the MELD score arouses our interest as a predictive model in estimating the long-term prognosis of CHD patients after PCI. To investigate this relation, a retrospective cohort study involving 5373 CHD patients undergoing PCI was carried out.

## 2. Methods

### 2.1. Study Design and Population

**Figure 1:**
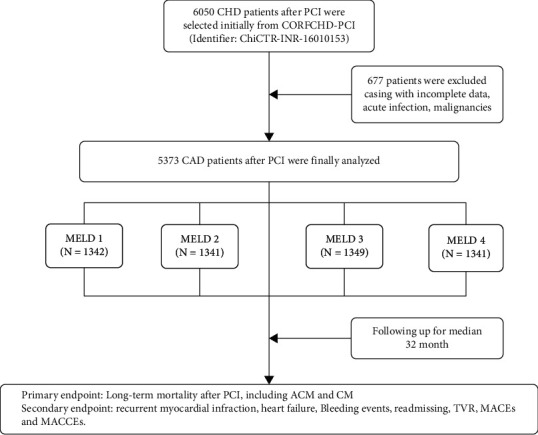
Flow chart of participant selection.

All the participants were recruited from the Clinical Outcomes and Risk Factors of Patients with Coronary Heart Disease after PCI (CORFCHDPCI) study, which was a large, single-center retrospective cohort study including 6,050 CHD patients who were admitted in the First Affiliated Hospital of Xinjiang Medical University from January 2008 to December 2016. The details of the design are registered on http://www.chictr.org.cn (identifier: ChiCTR-ORC-16010153). ►Figure 1 shows the flowchart of the inclusion and exclusion criteria in selection of participants. The inclusion criteria were CHD patients including non-ST-segment elevation acute coronary syndrome (ACS), ST-segment elevation ACS, and stable angina, who were undergoing coronary angiography, with stenosis ≥70%, and receiving at least one stent implantation. We excluded patients who had serious heart failure, rheumatic heart disease, valvular heart disease, congenital heart disease, pulmonary heart disease, and serious dysfunction of the kidney. A total of 677 patients were excluded casing with incomplete data, acute infection, and malignancies. Finally, 5373 patients were enrolled. This study complied with the Declaration of Helsinki, and the protocol was approved by the ethics committee of the First Affiliated Hospital of Xinjiang Medical University. Because of the retrospective design of the study, the need to obtain informed consent from eligible patients was waived by the ethics committee.

### 2.2. Clinical Data Collection

We collected the demographic data, clinical characteristics, risk factors, blood samples, biochemical parameters, electrocardiographs (ECG), echocardiography, coronary angiography, PCI procedure, and long-term outcomes for CHD patients who underwent PCI. The cardiovascular risk factors included smoking status, alcohol consumption, previously diagnosed diabetes, hypertension, and familial history of CHD. During the follow-up period, the use of *β*-blockers, angiotensin-converting enzyme inhibitors (ACEIs), angiotensin II receptor blockers (ARBs), statins, aspirins, clopidgrel, and calcium channel blockers (CCBs) was recorded.

### 2.3. Definition of Risk Factors

Hypertension was defined as the patient having a definite history of hypertension and on active treatment with antihypertensive drugs or with blood pressure measurements 140/90 mm Hg on at least three resting measurements on at least two separate health care visits according to the American Heart Association recommendations [10]. Diabetes mellitus was positive in patients with a definite history of diabetes and treatment with glucose-lowering agents or a fasting plasma glucose ≥7.1 mmol/L or 2-hour postload glucose ≥11.1 mmol/L [11]. The diagnostic criteria for hyperlipidemia were mainly obtained from the ‘Guideline of Chinese Adult Dyslipidemia Prevention and Treatment (2016)' [12]. Smoking status classifications were current smokers, former smokers, and never-smokers. Persons reporting regular tobacco use in the previous 6 months were considered current smokers. Persons who had ingested alcohol in the last 6 months were considered alcohol users.

### 2.4. Blood Sampling and Calculation of the MELD Score

All measurements of INR, sCr, and TB were performed at the presentation of the patients prior to the initiation of anticoagulant therapy and coronary angiography. The MELD score was calculated by using abovementioned three simple metrics including INR, sCr, and TB as follows: 3.8 × ln TBIL (mg/dL) + 11.2 × ln (INR) + 9.6 × ln Cr (mg/dL) + 6.4 × etiology value, and the etiology value is 0 for biliary or alcoholic cirrhosis or 1 for all others [13].

### 2.5. End Points

As described previously [14], the primary point was defined as the occurrence of long-term mortality, including all-cause mortality (ACM) and cardiac mortality (CM) during the median follow-up of 32 months. The secondary endpoints were major adverse cardiac events (MACEs), major adverse cardiac and cerebrovascular events (MACCEs), bleeding events, and readmission. Briefly, MACE was defined as the combination of cardiac death, myocardial reinfarction, and target vessel reconstruction (TVR), while MACCE was defined as MACE plus stroke. Reinfarction was defined according to the third universal definition of myocardial infarction [15]. Stroke was defined as an acute neurological deficit accompanied by brain imaging compatible with a recent event including hemorrhage, embolism, thrombosis, or aneurysm rupture, persisting for >24 hours. Target vessel revascularization (TVR) was defined as any repetitive revascularization of treated vessel with a stenosis of at least a 50% diameter in the presence of ischemic signs or symptoms or stenosis of at least 70% in the absence of ischemic signs or symptoms. Bleeding events were defined using the criteria of the Academic Research Consortium definition [16]. All incidents were determined by a committee that was blinded to the group of patients.

### 2.6. Follow-Up

All of the participants received regular follow-up after discharge at the end of 1 month, 3 months, 6 months, 1 year, 3 years, and 5 years. Overall, all of them were followed up for at least 2 years, and the longest follow-up time was 10 years. Trained investigators follow-up the patients by telephone contacts or office visits as necessary. The compliance of the drugs and adverse events was also assessed at every clinic visit.

### 2.7. Statistical Analyses

All analyses were performed using the SPSS 22.0 for Windows statistical software (SPSS Inc, Chicago, Illinois, United States). Continuous data were presented as the mean and standard deviation (SD) or median (interquartile range, IQR) according to the results of the normal test. Categorical data were expressed as the frequencies and percentages (%). The differences between normally distributed numeric variables were analyzed by ANOVA, while nonnormally distributed variables were analyzed by the Mann–Whitney *U* test. The chi-square test was employed for the comparison of categorical variables. Kaplan–Meier analysis was used for cumulative incidence rates of long-term outcomes, and the log-rank test was used to compare between groups. Multivariable Cox regression analysis was performed to assess the predictive value of the MELD for outcomes during and up to a 10-year follow-up. Hazard ratios (HRs) and 95% confidence intervals (CIs) were calculated. *p* value < 0.05 was considered significant.

## 3. Result

### 3.1. Basic Characteristics of Participants

A total of 6,050 patients were evaluated initially. Finally, 5373 patients were enrolled in this study. The patients were divided into four groups according to the MELD score: the MELD1 group (<0.66, *n* = 1342), MELD2 group (0.66–2.70, *n* = 1341), MELD3 group (2.70–4.75, *n* = 1349), and MELD4 group (>4.75, *n* = 1341). As shown in ► Table 1, age, smoking, female ratio, blood urea nitrogen (BUN), diabetes, alcohol drinking, creatinine (Cr), and triglyceride (TG) were significantly different among four MELD groups (all *p* < 0.05). We did not find a significant difference between these four groups in regards to therapy of calcium channel blocker, angiotensin-converting enzyme inhibitor or angiotensin receptor blocker, clopidogrel, aspirin or statins, systolic blood pressure (SBP) and diastolic blood pressure (DBP), total cholesterol (TC), low-density lipoprotein cholesterol (LDL-C), lipoprotein a (Lp (a), high-density lipoprotein cholesterol, apolipoprotein A1, and apolipoprotein B (all *p* < 0.05).

### 3.2. Clinical Outcomes

The univariate Cox regression analysis is displayed in ►Table 2. Briefly, the primary endpoints differed significantly across different MELD groups (all *p* < 0.05), but no significant difference observed of the secondary endpoints was observed in this study (all *p* < 0.05). The incidence rates of ACM in the MELD1, MELD2, MELD3, and MELD4 groups were 55 (4.1%), 66 (4.9%), 65 (4.8%), and 87 (6.5%), respectively (*p*=0.038). Meanwhile, the incidence rates of CM were reported as 42 (3.1%), 51 (3.8%), 53 (3.9%), and 72 (5.4%) in the MELD1, MELD2, MELD3, and MELD4 groups (*p*=0.027). Variables that were significant (*p* < 0.05) in univariate Cox models were entered into multivariate Cox regression analysis. As shown in ►Tables 3 and 4, after adjusting the variables such as age, gender, smoking, drinking status, and diabetes using a multiple Cox regression analysis model, MELD4 was still associated with ACM (adjusted HR:1.57, 95%CI 1.052–2.354, *p*=0.027) and CM (adjusted HR:1.434, 95%CI 1.003–2.050, *p* = 0.048), but no significant relation was observed in secondary endpoints when compared to MELD1 (all *p* > 0.05).

**Figure 2:**
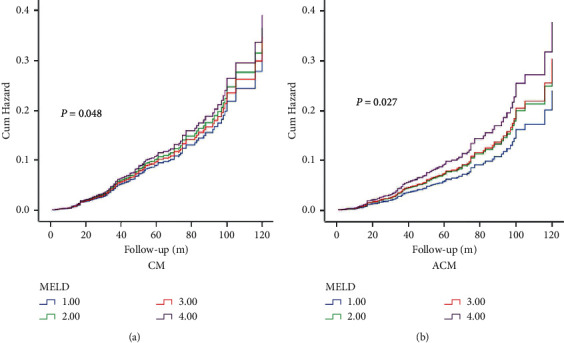
Cumulative Kaplan–Meier curve estimates of the time to the first adjudicated occurrence of primary and secondary endpoints.

As shown in ►Figure 2, the Kaplan–Meier curve indicated the cumulative risk for primary and secondary endpoints across different MELD groups. Significant differences of ACM and CM were observed in MELD groups. However, the secondary endpoints did not differ across the MELD groups (all *p* > 0.05).

## 4. Discussion

We firstly conducted a large cohort study to investigate the role of the MELD score in predicting the long-term outcome of CHD patients undergoing PCI. We observed remarked differences in the incidences of ACM and CM among different MELD groups, but no significant difference was found in secondary endpoints. After adjusting the age, gender, smoking, drinking status, and diabetes by a multiple Cox regression analysis, MELD4 was still associated with ACM and CM. Furthermore, then Kaplan–Meier curve indicated that there were significant differences in ACM and CM across four MEDLD groups.

The MELD score is initially used in predicting the prognosis of liver transplantation [4], which uses three simple metrics including INR and total bilirubin (TB), as well as serum creatinine, to quantify the degree of liver dysfunction [13]. Interestingly, it is reported that the component of the MELD score has a relation to the prognosis of various cardiovascular diseases. Recently, MELD scores have been used extensively to predict operative mortality in patients undergoing cardiac surgery [17, 18]. In fact, evidence shows that MELD is highly predictive of mortality in tricuspid surgical patients [19, 20]. An MELD > 15 score was associated with a nine-fold higher odds of mortality in emergency cardiac transplantation [21]. Furthermore, the MELD score plays an effective role in the prediction of nonoperative outcomes, such as evaluating risk for patients with heart failure [22, 23]. Also, the combining LVEF with the MELD score may be useful to predict long-term survival in patients with ACS who were undergoing PCI [9]. In this study, we observe that the MELD score is associated with the occurrences of ACM and CM, predicting a prognostic role for prognosis of CHD patients undergoing PCI. After adjusting the other confounding factors, participants in MELD4 groups still have an increased risk of 1.57-fold ACM and 1.43-fold CM, respectively. In accordance with the previous study [9], the fourth MELD score group had the highest risk of ACM or CM, implying that a higher MELD score is correlated with an increased mortality in CHD patients after PCI. These findings indicate that the MELD score could be used as a predictable tool for both the liver disease and cardiovascular disease.

The reasons for MELD in predicting the prognosis of CHD patients undergoing PCI may be explained as follows: First, chronic kidney diseases may affect the progression of CHD patients. It is reported that serum creatinine measured at hospital admission seems to be associated with mortality in patients with ACS [24]. Longer hospital stays and higher adverse events are reported in ACS with CKD patients [25, 26]. In the Credo-Kyoto study, statin therapy was associated with a lower incidence of MACE in patients with GFR ≥30–60, but not in patients with more severe CKD. Mild-to-moderate chronic kidney diseases have a predicative effect on one-year outcomes after PCI [27]. Impaired renal function has been established as a significant and independent predictor of adverse cardiovascular events among patients admitted for STEMI receiving PCI [28].

Second, TB may play dual roles in the progress of CHD patients. In the PRIME study, which has described the relationship of serum bilirubin levels and cardiovascular risk as a U-shaped curve, implies that bilirubin exerts a protective effect, yet excessive concentrations may have a detrimental effect [29]. Instead, TB levels are reported to be independently associated with high SYNTAX score and, thus, may reflect the severity of NSTEMI [30]. In another study, initial TB was a powerful prognostic marker, which can improve prediction of in-hospital MACE in patients with STEMI undergoing primary PCI with DES [31]. Higher serum TB is still independently associated with in-hospital adverse events in patients with STEMI who underwent primary PCI, although serum TB is measured after primary PCI [32]. The mechanism may be that TB involves the post-PCI coronary no-reflow and, thus, increases the in-hospital MACEs [33]. Generally, higher TB is related to a worse outcome of CHD participants with PCI intervention, which is line with our findings.

Third, it has recently been shown that an increased INR in the absence of anticoagulant therapy is associated with mortality in patients with both acute pulmonary embolism (PE) and heart failure [8, 34]. Okada showed an increased INR was independent predictor of all-cause mortality in acute heart failure patients without anticoagulant therapy [10]. Similarly, an elevated INR is positively associated with mortality in patients with prevalent CHD not on oral anticoagulant therapy [35]. The mechanism may be accounted for that increased INR is not only associated with activated coagulation but also represents a serious inflammatory state in ACS [34], which may worsen the prognosis of CHD patients after reperfusion.

Finally, different studies have different cut-off values of the MELD score. In CABG patients grouped into low (<9), moderate (9–14), and high (≥15) MELD classifications, an elevated MELD score displays a higher risk of perioperative morbidity and mortality [36]. Meanwhile, another study indicates that mild-to-moderate chronic heart failure patients with MELD scores ≥10 had a significantly higher incidence of cardiac death than those with MELD scores <10 [37]. Evidence shows that the ACS patients undergoing PCI were divided into two subgroups based on the cut-off point of the MELD score; low (≤7.3) and high subgroups (>7.3), the cardiac death (5.0% vs. 1.5%, *p* < 0.001), and all-cause total mortality (14% vs. 18%, *p* < 0.001) are higher in patients with high MELD score than those with low MELD score [9]. Overall, independent of different cut-off values of MELD scores applied, the higher MELD score can be considered as a good predictor of CHD patients with PCI treatment.

Our study has some advantages over previous studies. First, the MELD score seems to present advantages and is more accessible in a clinical setting through simple common laboratory values. Second, this is a large cohort study which involves 5373 participants with a 10-year follow-up. However, some limitations should also be strengthened in this study. First, we failed to monitor the inflammatory or neurohumoral markers, including C-reactive protein, brain natriuretic peptide, angiotensin II, and norepinephrine levels, which may better explain the higher MELD values increased in patients with a malignant mechanism and, thus, enhanced the risk of cardiac events. Second, the present study was a single retrospective cohort design, and the findings may not be generated to other population. Herein, our results must be further verified in a multicenter, prospective study to confirm the association between MELD scores and adverse outcomes in CHD patients undergoing PCI treatment.

## 5. Conclusions

We found that higher MELD score was associated with ACM and CM, predicting a prognostic role for CHD patients undergoing PCI, expanding the utilization of the MELD score from the liver to heart. The MELD score is a simplified risk model and, thus, will help predict the prognosis of patients undergoing PCI, which is not currently accounted for other risk models.

## Figures and Tables

**Table 1:** Characteristics of participants of the two groups.

Variables	MELD integral quartile						
	MELD 1.0 *n* (%)	MELD 2.0 *n* (%)	MELD 3.0 *n* (%)	MELD 4.0 *n* (%)	Total *n* (%) or mean	Chi-square or F	*p* value
Age, years	59.25 ± 10.23	58.41 ± 10.75	59.61 ± 10.88	60.79 ± 11.20	59.51 ± 10.80	11.311	<0.001
Female *n* (%)	621 (46.3%)	341 (25.4%)	249 (18.5%)	177 (13.2%)	1388 (25.8%)	442.798	<0.001
Smoking, *n* (%)	413 (30.8%)	517 (38.6%)	594 (44.0%)	625 (46.6%)	2149 (40.0%)	82.289	<0.001
Alcohol drinking, *n* (%)	313 (23.3%)	380 (28.3%)	433 (32.1%)	440 (32.8%)	1566 (29.1%)	36.872	<0.001
Diabetes, *n* (%)	368 (27.4%)	320 (23.9%)	294 (21.8%)	330 (24.6%)	1312 (24.4%)	11.844	0.008
Hypertension, *n* (%)	595 (44.3%)	545 (40.6%)	584 (43.3%)	588 (43.8%)	2312 (43.0%)	4.46	0.226
SBP, mmHg	128.16 ± 18.34	127.05 ± 18.86	126.88 ± 18.60	126.33 ± 19.25	127.11 ± 18.77	2.241	0.081
DBP, mmHg	76.56 ± 11.07	76.33 ± 11.10	76.46 ± 11.42	75.70 ± 11.54	76.26 ± 11.29	1.571	0.194
BUN, mmol/L	5.01 ± 1.47	5.39 ± 1.48	5.60 ± 1.56	6.11 ± 1.91	5.53 ± 1.66	108.233	<0.001
GLU, mmol/L	6.72 ± 3.41	6.55 ± 2.89	6.41 ± 3.12	6.59 ± 3.08	6.57 ± 3.13	2.248	0.081
TG, mmol/L	1.99 ± 1.37	1.92 ± 1.37	1.87 ± 1.18	1.84 ± 1.19	1.90 ± 1.28	3.636	0.012
TC, mmol/L	3.99 ± 1.08	3.95 ± 1.07	3.97 ± 1.15	3.94 ± 1.13	3.96 ± 1.11	0.553	0.646
LDL-C, mmol/L	2.46 ± 0.88	2.47 ± 0.90	2.47 ± 0.97	2.48 ± 0.91	2.46 ± 0.92	0.176	0.913
HDL-C, mmol/L	1.01 ± 0.44	1.02 ± 0.52	1.04 ± 0.50	1.01 ± 0.46	1.02 ± 0.48	0.817	0.484
ApoA1, mmol/L	1.16 ± 0.31	1.17 ± 0.33	1.17 ± 0.32	1.16 ± 0.31	1.17 ± 0.32	0.509	0.676
ApoB, mmol/L	0.85 ± 0.33	0.86 ± 0.42	0.86 ± 0.44	0.84 ± 0.37	0.85 ± 0.39	0.786	0.502
Lp (a), mmol/L	227.95 ± 184.33	227.11 ± 188.82	215.03 ± 164.59	212.86 ± 164.59	220.75 ± 177.76	2.586	0.051
EF (%)	61.37 ± 7.03	60.96 ± 6.95	61.15 ± 6.94	60.96 ± 7.14	61.11 ± 7.02	0.919	0.431
LVEDD, mm	49.81 ± 5.44	49.99 ± 5.49	49.84 ± 5.58	50.17 ± 5.63	49.95 ± 5.53	1.038	0.375
CCB, *n* (%)	157 (11.8%)	166 (12.4%)	160 (11.9%)	138 (10.4%)	621 (11.6%)	3.049	0.384
*β*-Blocker, *n* (%)	515 (38.6%)	557 (41.7%)	523 (38.9%)	567 (42.5%)	2162 (40.4%)	6.345	0.092
ACEI or ARB,n (%)	301 (22.6%)	312 (23.4%)	292 (21.7%)	305 (22.9%)	1210 (22.7%)	1.14	0.767
Cr, umol/L	58.49 ± 10.68	70.57 ± 10.26	78.65 ± 11.80	94.97 ± 21.77	75.67 ± 19.58	1511.004	<0.001
Statins, *n* (%)	690 (52.0%)	743 (55.9%)	733 (54.7%)	727 (54.7%)	2893 (54.3%)	4.44	0.218
New-generation stent, *n* (%)	1260 (93.9%)	1260 (94.0%)	1275 (94.6%)	1266 (94.4%)	5061 (94.2%)	0.849	0.838
CTO, *n* (%)	281 (20.9%)	299 (22.3%)	306 (22.7)	364 (27.1%)	1250 (23.3%)	16.312	0.001
ML, *n* (%)	851 (63.4%)	874 (65.2%)	860 (63.8%)	913 (68.1%)	3498 (65.1%)	7.945	0.047

ACEI, angiotensin-converting enzyme inhibitor; ApoA1, apolipoprotein A1; ApoB, apolipoprotein B; ARB, angiotensin receptor blocker; BMI, body mass index; BUN, blood urea nitrogen; CCB, calcium channel blocker; Cr, creatinine; CTO, chronic total occlusion lesions; DBP, diastolic blood pressure; EF, ejection fraction; GLU, glucose; HDL-C, high-density lipoprotein cholesterol; LVEDD, left ventricular end-diastolic dimension; LDL-C, low-density lipoprotein cholesterol; Lp (a), lipoprotein a; MELD, model for end-stage liver disease; ML, multivessel lesions; SBP, systolic blood pressure; TC, total cholesterol; TG, triglyceride; UA, uric acid. *Note.* The boldfaced values indicate *p* < 0.05.

**Table 2:** Outcome comparison between groups.

Outcomes	MELD1 (*n* = 1342)	MELD2 (*n* = 1341)	MELD3 (*n* = 1349)	MELD4 (*n* = 1341)	*χ*2	*p*
ACM, *n* (%)	55 (4.1)	66 (4.9)	65 (4.8)	87 (6.5)	8.452	0.038
CM, *n* (%)	42 (3.1)	51 (3.8)	53 (3.9)	72 (5.4)	9.175	0.027
MACCE, *n* (%)	172 (12.8)	189 (14.1)	194 (14.4)	209 (15.6)	4.262	0.234
MACE, *n* (%)	159 (11.8)	172 (12.8)	178 (13.2)	185 (13.8)	2.386	0.496
Heart failure, *n* (%)	44 (3.3)	43 (3.2)	38 (2.8)	38 (2.8)	0.808	0.848
Bleeding events, *n* (%)	41 (3.1)	29 (2.2)	43 (3.2)	46 (3.4)	4.288	0.232
Readmission, *n* (%)	175 (13.0)	184 (13.7)	182 (13.5)	196 (14.6)	1.493	0.684
TVR, *n* (%)	67 (5.0)	67 (5.0)	82 (6.1)	66 (4.9)	2.505	0.474

MELD, model for end-stage liver disease; ACM, all-cause mortality; CM, cardiac mortality; MACE, major adverse cardiovascular event; MACCE, major adverse cardiovascular and cerebrovascular event; TVR, target vessel reconstruction.

**Table 3:** Multivariable Cox regression analyses of ACM.

Variables	Wald	HR	ACM 95%CI	*p*
Age	7.056	1.02	1.005–1.032	0.038
Sex	0.04	1.036	0.732–1.476	0.842
Man		[1] (reference)		
Women				
Smoke	0.901	0.84	0.595–1.198	0.342
Drink wine	0.204	1.09	0.756–1.565	0.652
DM	0.624	1.13	0.833–1.535	0.43
TG	0.046	1.01	0.911–1.124	0.829
				0.168
** **1		[1] (reference)		
** **2	1.004	1.24	0.817–1.870	0.316
** **3	1.241	1.27	0.835–1.924	0.265
** **4	4.859	1.57	1.052–2.354	0.027

**Table 4:** Multivariable Cox regression analyses of CM.

Variables	Wald	HR	95%CI	*p*
Age	18.568	1.027	1.014–1.039	<0.001
Sex	0.016	1.020	0.747–1.392	0.9
Man		[1] (reference)		
Women				
Smoking	0.092	0.953	0.699–1.300	0.762
Drinking	0.029	1.029	0.743–1.424	0.864
DM	0.132	1.053	0.798–1.388	0.716
TG	1.022	1.046	0.959–1.140	0.312
*MELD score*				
** **1	4.059	[1] (reference)		
** **2	1.121	1.217	0.846–1.750	0.29
** **3	0.746	1.177	0.813–1.705	0.388
** **4	3.897	1.434	1.003–2.050	0.048

## Data Availability

Data are available on request.
